# Contextual factors in the surveillance of non-communicable diseases (NCD) in Germany: political, social and environmental indicators

**DOI:** 10.25646/14104

**Published:** 2026-04-29

**Authors:** Laura Neuperdt, Oktay Tuncer, Rebekka Mumm, Susanne Jordan, Anne Starker, Kristin Manz, Stephan Müters, Christin Heidemann

**Affiliations:** 1 Robert Koch Institute, Department of Epidemiology and Health Monitoring, Berlin, Germany; 2 WIG2 GmbH – Scientific Institute for Health Economics and Health System Research in Leipzig, Germany

**Keywords:** Diabetes mellitus, Type 2 diabetes, Health expenditure, Non-communicable diseases, Population health, Tobacco control, Health policy, Taxes, Poverty, At-risk-of-poverty rate, Consumer price index, Inflation rate, NCD Surveillance

## Abstract

**Background:**

The health of the population is shaped by political, social and environmental conditions – referred to as contextual factors in this paper – which influence the risk of type 2 diabetes and other non-communicable diseases (NCD). The article provides an overview of selected indicators for this topic.

**Method:**

The indicators were developed based on a literature review and a multi-stage selection process, considering their relevance for the prevention of diabetes and other NCD as well as the availability of data. Available nationwide data were used for operationalisation.

**Results:**

Six indicators were agreed upon: tobacco control, the consumer price index, food taxation and prevention expenditure in the area of health policy measures, the at-risk-of-poverty rate in the area of employment and social affairs, and use of means of transport in the area of built and physical environment. The time trends for these indicators are integrated into the NCD Surveillance of the Robert Koch Institute (RKI) and are presented on the web portal of the federal health reporting. The results for all indicators point to inadequate prevention measures.

**Conclusions:**

Context indicators help to track changes over time in environmental determinants of health. As part of NCD Surveillance at the RKI, this topic will be continuously developed.

## 1. Introduction

Non-communicable diseases (NCD) are responsible for the premature death of around 18 million people worldwide before the age of 70 [1]. The causes of NCD are complex and, in addition to biological factors and individual behaviour, are linked to living conditions. Many of these causes can be influenced by targeted measures. The World Health Organisation’s (WHO) Global Action Plan for the Prevention and Control of NCD therefore identifies surveillance as a key tool for reducing the burden of disease caused by NCD [2]. Surveillance is understood as the continuous, systematic collection, analysis and interpretation of health-related data [3, 4], in order to create an up-to-date and comprehensive information base on which stakeholders from politics and the health sector can develop strategies for improved prevention and care of NCD. Modern surveillance of NCD in the sense of the ‘Health in All Policies’ approach requires the integration of social, political and environmental determinants of health [5].

The National Diabetes Surveillance research project at the Robert Koch Institute (RKI) established a comprehensive system between 2015 and 2024 to provide an indicator-based overview of the epidemiology of diabetes in Germany. This system served as a pilot project for the current development of a comprehensive NCD Surveillance system and its determinants at the RKI. Through a multi-stage research and selection process and using selection criteria, 40 indicators or indicator groups were agreed upon within the framework of Diabetes Surveillance for four fields of action [6, 7]. In the field of action ‘Reducing the risk of disease’ (focus on primary prevention), the topic contextual factors was also agreed upon during the initial indicator selection process; however, no indicators could be identified for this topic, as no representative data for Germany was available for evaluation regarding the researched topics (e.g. walkability of the residential environment) [6]. Given the central importance of this topic for the surveillance of diabetes and of NCD, it was decided to agree on indicators for these topics through an in-depth literature search and a separate selection process.

In the following, contextual factors are understood as all conditions in which people live and work and that may influence their health, health behaviour and quality of life [2]. Such conditions, which include political, social and environmental factors, have an impact on the risk factors and the incidence of NCD.

At the global level, the WHO defines evidence-based and cost-effective environmental prevention measures for the prevention of NCD through its so-called ‘Best Buys’ [8], with a focus on reducing tobacco and alcohol consumption and promoting healthy diets and physical activity. These approaches are also reflected in European frameworks that capture the political and regulatory context of health-related behaviours, such as the Healthy Food Environment Policy Index (Food EPI) [9] and the Physical Activity Environment Policy Index (PA-EPI) [10].

Furthermore, environmental prevention measures that address the framework conditions can yield simultaneous benefits across multiple areas, e.g. both improving public health and having positive effects on the climate and the natural environment. These are referred to as co-benefits [11]. For example, a shift from motorised private transport to active forms of mobility, such as walking or cycling, increases physical activity and reduces both the risk of developing multiple NCD and greenhouse gas emissions [12].

The monitoring of contextual factors using relevant indicators as part of a surveillance programme makes it possible to systematically capture environmental factors, thereby identifying prevention needs and initial points for health-promoting changes in people’s living environments to reduce the risk of NCD in the population.

**Key messages**

► Through successive rounds of literature search and selection, six contextual factors relevant to the risk of type 2 diabetes and other NCD were agreed upon as indicators.
► In 2021, Germany was among the five countries with the lowest tobacco control measures compared to other European countries.
► Since 2015, prices for food and non-alcoholic beverages have risen significantly – the health value of food is not considered in taxation.
► At 5 % in 2023, the share of prevention expenditure in total health expenditure is relatively low.
► The at-risk-of-poverty rate has risen since 2005, reaching 16.2 % in 2024.
► In 2023, for around half of all distances covered (51 %), the car (as driver or passenger) was used as the main mean of transport.

This paper describes how indicators for contextual factors were selected, focusing on primary prevention within Diabetes Surveillance. The selected indicators were subsequently adopted into a comprehensive NCD Surveillance system. Current data are reported for the selected indicators.

## 2. Methods

**Figure 1:**
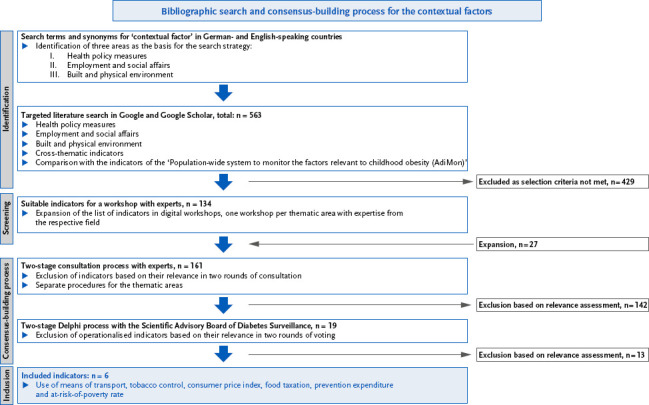
Flow Chart

The selection and definition of indicators for contextual factors were carried out in several steps, drawing on the process previously used to agree on indicators for Diabetes Surveillance [6, 13]. These steps are described in more detail below (Figure 1).

### 2.1 Research regarding the indicators

Existing surveillance systems for diabetes and NCD (including those from Switzerland [14], Canada [15] and Australia [16]) were reviewed and served as a guide for the development of indicators relating to contextual factors. It was found that there is no standardised terminology for this topic in German-speaking countries as opposed to the English-speaking countries where the term ‘contextual factors’ is frequently used in a public health context.

In parallel with the research process, three key areas were derived from the public health framework of the National Institute for Health and Care Excellence (NICE) [17] to further define the topic: ‘health policy measures’, ‘employment and social affairs’ and ‘built and physical environment’. The framework concept views health as the result of a complex system of multiple and partly interacting factors, known as health determinants. In addition to genetic and biological factors, these primarily include social, economic, political and environmental aspects.

A precise set of search terms was subsequently defined for the three topics mentioned, as well as for the overarching concept of ‘contextual factors’. This set was used in Google and Google Scholar to conduct a targeted literature search, including grey literature (Supplementary material Table 1). The selection and refinement of the search terms took place iteratively on the basis of initial searches and internal discussions, with all search strings being narrowed down in terms of content and formulated in both English and German. In this step, indicators from the existing indicator system of the RKI’s project ‘Population-wide system to monitor the factors relevant to childhood obesity (AdiMon)’ [18] were also included. The result of the research process yielded a list of 563 indicators (Figure 1).

### 2.2 Selection of indicators

The following criteria, which had already been considered during the consensus-building process for the Diabetes Surveillance indicators, were applied in the subsequent selection process for the contextual factors:

► Relevance to Diabetes Surveillance and, in the future, to NCD Surveillance,
► Modifiability of the indicator, i.e. potential for change through policy measures,
► Unambiguity of the indicator, i.e. the indicator is clearly defined, measurable and unambiguous,
► Data availability.

In addition, the following criteria had to be met for the aim of integrating context factors into the field of action ‘Reducing the risk of disease’:

► environmental indicator,
► Evidence that the indicator reflects a risk factor for diabetes and other NCD.

In the first selection stage, 429 indicators that did not meet the specified criteria were excluded (Figure 1). The remaining 134 indicators were discussed in interdisciplinary workshops involving experts from academia (university and non-university research), the public health service (federal and state authorities) and professional associations. To take account of the large number and heterogeneity of the indicators identified, three workshops were held, with one workshop dedicated to each of the three areas: ‘health policy measures’, ‘employment and social affairs’ and ‘built and physical environment’. Due to the COVID-19 pandemic, the workshops were conducted online. During the workshops, 27 indicators were added. Subsequently, the experts in the workshops assessed the indicators for their respective topics using a two-stage Delphi procedure, evaluating their relevance for Diabetes Surveillance with a focus on prevention (action area ‘Reducing the risk of disease’). To do this, they used a nine-point scale (0: no relevance, 9: high relevance) in written questionnaires. In addition, the experts were able to indicate whether they were aware of a data source for the respective indicator and had the opportunity to provide comments in designated comment fields. An accompanying discussion was facilitated via the group forum of the ‘Basic Support for Cooperative Work – Informations Technik Server Bund (BSCW-ITZBund)’ server during an initial round of consultation and via email exchange during a second round of consultation. Indicators with an average relevance rating in the lower or middle range (median < 7) were excluded. Of the 161 indicators, 19 were agreed upon following the relevance assessment for the final consultation process with the Scientific Advisory Board of Diabetes Surveillance (Supplementary material Table 2).

As with the previous process of selecting all existing Diabetes Surveillance indicators, the members of the Scientific Advisory Board assessed these selected and operationalised indicators using a Delphi method to evaluate their relevance for Diabetes Surveillance. To this end, two consecutive rounds were conducted, each comprising an online discussion and a vote via a written questionnaire. Indicators that received a high relevance rating (median 7 – 9 on a 9-point scale) in the second round of voting were considered to have achieved consensus. In total, this applied to six indicators, which were included in the Diabetes Surveillance indicator set (Figure 1).

The Diabetes Surveillance project concluded in December 2024 in favour of further development into a comprehensive NCD Surveillance system. Data on the selected indicators were documented in PDF fact sheets at the end of the Diabetes Surveillance project [19]. Furthermore, all selected indicators have been incorporated in the indicator set of the NCD Surveillance under the ‘Contextual Factors’ section; their results, methodology and classification are continuously presented and updated on the RKI’s health reporting web portal [20].

## 3. Results

**Figure 2:**
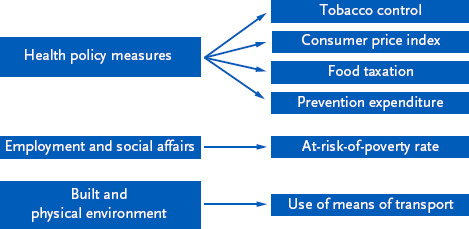
Areas of contextual factors and agreed indicators

As a result of the consensus-building process, the following six indicators were operationalised: **tobacco control**, **consumer price index**, **food taxation** and **prevention expenditure** in the area of health policy measures, **at-risk-of-poverty rate** in the area of employment and social affairs, and **use of means of transport** in the area of the built and physical environment (Figure 2). The significance of the individual indicators is outlined below, and selected results are described.

### 3.1 Health policy measures

Preventive measures are among the health policy measures designed to protect the population and reduce health risks. Many preventive measures are regulated in the Social Security Codes and financed by the health system [17, 21]. In this context, the agreed indicators each reflect different aspects. **Tobacco control**, as measured by the Tobacco Control Scale, serves to evaluate measures aimed at reducing tobacco consumption and thus contributes to the protection of the population [22]. The **consumer price index** and **food taxation** can influence household purchasing behaviour [23], and **prevention expenditure** reflects services designed to prevent the onset or spread of diseases in advance or at an early stage [24].

#### Tobacco control

**Figure 3:**
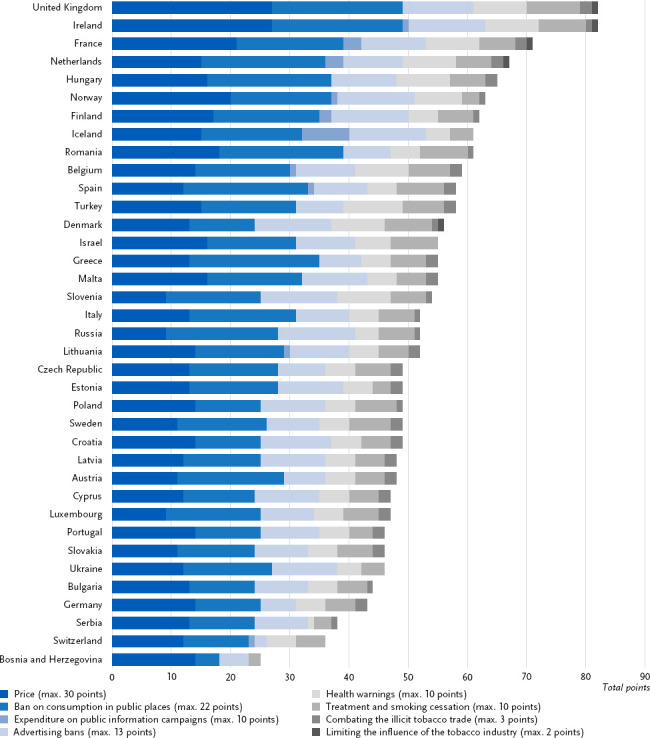
Total score derived from the scores of the components of the 2021 Tobacco Control Scale in a country comparison. Source: Tobacco Control Scale [27]

Smoking cigarettes and other tobacco products increases the risk of developing type 2 diabetes and other NCD such as lung cancer, chronic obstructive pulmonary disease (COPD) and cardiovascular diseases [25]. Tobacco control measures aim to reduce the proportion of smokers in the population and ensure comprehensive protection against exposure to second-hand smoke [26]. The European Tobacco Control Scale developed by Joossens et al. [22] quantifies the implementation of legislative measures using a points system at the level of individual European countries, thereby enabling international comparison [27]. A total of 100 points can be achieved across a current total of eight policy areas (Figure 3). A higher total score indicates that a country has implemented more comprehensive and effective measures to curb tobacco consumption and second-hand smoke. The points distribution system for the policy areas has changed slightly since 2019 due to the addition of new dimensions within the policy areas [28].

With a score of 43 points on the 2021 Tobacco Control Scale, Germany has one of the lowest overall scores in Europe (Figure 3) and has steadily slipped from 22nd (out of 30 countries) in 2005 to 34th place (out of 37 countries) in 2021 [27]. During this period, improvements were observed only in the areas of ‘public place bans’ and ‘advertising bans’, with scores rising from 2 to 11 points (out of a possible 22) and from 4 to 6 points (out of a possible 13) respectively. In the ‘price’ area, there was a decrease from 20 to 14 points (out of a possible 30), whilst in the ‘treatment for smoking cessation’ and ‘health warnings’ areas, with 5 points each (out of a possible 10), there were hardly any changes. Whilst ‘illicit tobacco trade’ was represented on the scale with 2 points (out of a possible 3), no points were awarded for the areas ‘spending on public information campaigns’ (out of a possible 10) and ‘tobacco industry interference’ (out of a possible 2) [27].

#### Consumer price index

The consumer price index, measures the ‘average price trend of all goods and services purchased by private households for consumption purposes’ [29] compared to a defined base year (currently 2020). A distinction is made between consumption categories (e.g. food) and sub-categories (e.g. vegetables, meat and meat products) [29]. As food prices can influence purchasing behaviour, particularly in lower-income households [30], it is important to consider the consumer price index for selected food groups for which a link to diabetes and other NCD has been observed [31, 32]. The food groups selected were fruit, vegetables, confectionery, mineral water, ready meals, meat and meat products, and sugar-sweetened beverages (excluding mineral water). For comparison, the consumer price index for all food and non-alcoholic beverages is shown.

**Figure 4:**
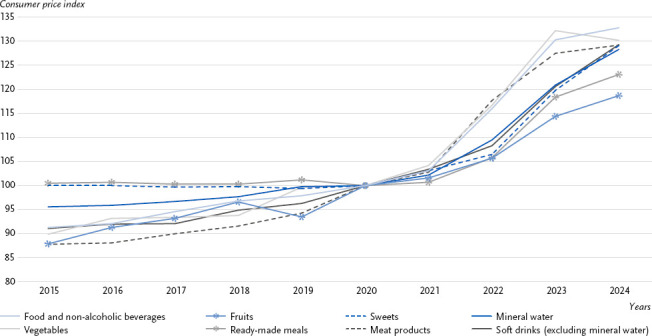
Trend in the consumer price index between 2015 and 2024 (index base 2020 = 100). Source: Consumer Price Index (CPI) [29]

Between 2020 and 2024, the price increase for all food and non-alcoholic beverages (32.8 %) was higher than between 2015 and 2020 (9.5 %). Compared with the previous year, the price increase, also known as the inflation rate, for all food and non-alcoholic beverages in 2024 (1.9 %) was lower than in 2023 (12.3 %) and 2022 (12.5 %). Since 2015, prices for vegetables (44.8 %) and fruit (by 35.0 %), the regular consumption of which is considered protective against various NCD, have risen more significantly than those for readymade meals (by 22.5 %) and sweets (by 29.1 %), the regular consumption of which is considered to increase the risk of NCD (Figure 4) [33, 34].

#### Food taxation

In Germany, food is taxed via statutory value added tax (VAT) rates, which have a direct impact on food prices. As described above, food prices can influence purchasing behaviour [30].

In Germany, the taxation of food and drink is independent of their health benefits. Since 1990, the reduced VAT rate of 7 % has applied to all food products, including fruit, vegetables, sweets and meat products [35]. The standard VAT rate applies to drinks, including mineral water and sugar-sweetened beverages. The standard VAT rate has risen gradually from 14 % in 1990 to 19 % in 2007 and has remained constant since then, except for a temporary reduction to 16 % during the COVID-19 pandemic (July to December 2020) (Supplementary material Figure 1).

#### Prevention expenditure

Prevention aims to prevent or delay the onset, spread and consequences of diseases through targeted measures [36]. Expenditure on prevention provides an important indicator for assessing the level of investment in preventive measures. Information on nationwide health expenditure is provided by the Health Expenditure Accounts (GAR) of the Federal Statistical Office [24]. The item ‘prevention and health protection’ covers healthcare services aimed at preventing diseases in advance or at an early stage, or at curbing their spread [24].

In 2023, € 298 per person was spent on prevention and health protection in Germany. This corresponds to approximately 5.0 % of total health expenditure. The share of prevention expenditure in total health expenditure remained relatively constant between 1992 and 2020 (1992: 3.7 %; 2020: 3.7 %). In 2021 (7.2 %) and 2022 (8.5 %), there was an increase (Supplementary material Figure 2), which is attributable to expenditure on vaccinations and tests during the COVID-19 pandemic [37]. In 2023, the share of prevention expenditure in total health expenditure declined (5.0 %), thereby returning to pre-pandemic levels.

### 3.2 Employment and social affairs

For ‘employment and social affairs’, the research focused on work- and occupation-related and social contextual factors, as well as workplace health promotion, which are closely linked to risk factors for diabetes and other NCD [17, 38, 39]. For this area, the **at-risk-of-poverty rate** was finally agreed upon as the only indicator and therefore reflects only one aspect.

#### At-risk-of-poverty rate

Income plays an important role as a socio-economic factor influencing health. A low income is associated with an increased risk of developing type 2 diabetes or other NCD [40, 41]. The at-risk-of-poverty rate refers to the proportion of the population living under the at-risk-of-poverty threshold. To calculate the at-risk-of-poverty threshold, the average annual income of a household is adjusted for household size and age (equivalised disposable income) [42]. The at-risk-of-poverty threshold is considered to have been breached if this equivalised disposable income amounts to less than 60 % of the median income of the total population. People with an income of less than 60 % of the median income are therefore considered to be at risk of poverty. The at-risk-of-poverty rate in Germany is reported, among other things, using data from the annual microcensus [43].

**Figure 5:**
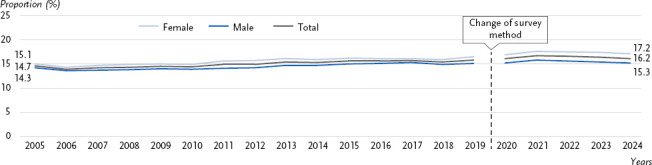
Trend in the at-risk-of-poverty rate (%) between 2005 and 2024 by gender. Source: Microcensus [46] There was a break in the time series in the official statistics for the 2020 survey year. The EU-SILC survey, which had previously been conducted separately, was integrated into the microcensus as a sub-sample. Due to the associated methodological changes, a direct comparison of the data from the 2020 survey year with previous years is no longer meaningful [47].

In 2024, 16.2 % of the population in Germany lived below the at-risk-of-poverty threshold. At 17.2 %, the proportion was higher for women than for men (15.3 %). Between 2005 and 2019, the proportion of women and men at risk of poverty has risen, slightly more sharply for women (from 15.1 % to 16.6 %) than for men (from 14.3 % to 15.2 %) (Figure 5). For the period from 2020 to 2024, the data do not initially indicate any further increase [44]. People under the age of 25 and those aged 65 and over are more likely to live below the at-risk-of-poverty threshold than those aged 25 to 64. Furthermore, a marked gradient can be observed across educational groups: at 38.3 %, people with a low level of education are significantly more likely to be at risk of poverty than those with a medium (15.6 %) or high level of education (7.4 %) [45].

### 3.3 Built and physical environment

The built and physical environment encompasses numerous health-related factors, including the quality of the residential and living environment and the traffic situation. Health-promoting urban planning that supports active forms of mobility – for example, through the targeted expansion of cycle paths and footpaths – has been shown to contribute to a reduction in the development of obesity and the risk of type 2 diabetes and other NCD by increasing physical activity [48, 49]. The indicator **‘use of means of transport’**, which has been selected for this area, reflects only one aspect of this.

#### Use of means of transport

Physical inactivity, i.e. a lack of exercise in various areas of life such as leisure, work, household chores and when covering everyday distances, represents a significant risk factor for the development of type 2 diabetes and other NCD [49–51]. The WHO recommends that adults be physically active for at least 150 minutes per week [52]. Physical activity whilst travelling can make an important contribution to achieving this recommended amount of exercise [53]. As the choice of transport mode also depends on environmental factors such as well-developed and attractive cycle paths and footpaths [54], use of means of transport is used as a proxy indicator for the built environment in the transport sector. The Mobility in Germany (MiD) study is a nationwide survey of households regarding their transport behaviour and records the percentage distribution (shares) of the individual means of transport primarily used by the population to cover their journeys, relative to the total volume of traffic (modal split) [55]. For each distance covered, all means of transport used were recorded. If a single mode of transport was mentioned, this was the main mean of transport; if several modes of transport were mentioned, the main mean of transport was the one used to cover the longest distance.

**Figure 6:**
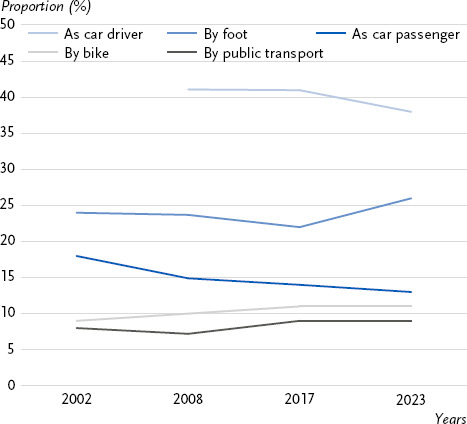
Trend over time in the proportion of journeys made using different main modes of transport, in % between 2002 and 2023. Source: Mobility in Germany [57]

In 2023, 40 % of distances were covered primarily by car (38 %) or truck (2 %) as the driver, and 13 % as a passenger in a car. A total of 26 % of distances were covered primarily by foot, 11 % by bicycle and 9 % by local public transport [56]. Between 2002 and 2023, motor vehicles (cars and trucks) remained the most frequently used means of transport for journeys. However, the proportion of distances covered by car as a passenger declined between 2002 (18 %) and 2023 (13 %). Looking at the trend over time, the proportion of distances covered by foot decreased from 24 % in 2002 to 22 % in 2017, before rising again to 26 % in 2023. In contrast, the use of the bicycle as the main mean of transport has increased from 9 % (2002) to 11 % (2023). The proportion of distances covered primarily by public transport fell slightly from 8 % in 2002 to 7 % in 2008, before rising again to 9 % in 2017 and 2023 (Figure 6). The use of transport varied significantly between age groups: people aged 40 to 49 predominantly used the car (51 %). Children (aged 7 – 10) and the very elderly (aged 80 and over) were most likely to walk (33 % and 37 % respectively). Young people aged 14 to 17 preferred to use public transport (27 %) and travel by car as passengers (25 %) [56].

## 4. Discussion

As part of the process of selecting indicators for the topic ‘contextual factors’ of the National Diabetes Surveillance at the RKI, six indicators (tobacco control, consumer price index, food taxation, prevention expenditure, at-risk-of-poverty rate and use of means of transport) were agreed upon and operationalised. These indicators complemented the existing set of indicators of the National Diabetes Surveillance and provide information on environmental factors, such as political, social and environmental conditions. Available data from official statistics, health expenditure accounts and population surveys as well as relevant legislation were used as data sources. This allows the reporting of trends over time, and, in some cases, of results stratified by age, gender or education. The indicators were subsequently incorporated into a comprehensive NCD Surveillance system at the RKI, the results of which are visualised on the web portal of the federal health reporting.

### 4.1 Indicator selection process

Drawing on experience gained during the development phase of the Diabetes Surveillance [6], the selection of contextual factors was carried out in a structured, multi-stage process, involving experts and the Diabetes Surveillance Scientific Advisory Board. This ensured the substantive quality and public health relevance of the selected indicators. One challenge was that the inconsistent definition of the term ‘contextual factors’ identified in the initial review of existing surveillance systems for diabetes and NCD made a systematic literature search for suitable indicators difficult; consequently, a snowball sampling approach had to be used for searches on Google and Google Scholar. The literature search on the topic of contextual factors included search terms from the areas of health policy measures, employment and social affairs, and the built and physical environment. The lack of available data for reporting time series and current nationwide representative values prevented the inclusion of further contextually relevant indicators.

### 4.2 Results of the indicators

Overall, the current status of the agreed indicators for contextual factors confirms that an expansion of environmental prevention measures in line with ‘Health in All Policies’ [45] is necessary to reduce NCD [58].

In terms of tobacco control, as measured by the Tobacco Control Scale, Germany ranks among the lowest in Europe. Across all areas of tobacco control policy, there is potential in Germany for more consistent implementation of measures to reduce smoking rates and exposure to second-hand smoke among the population [58–60]. The Tobacco Control Scale uses a points system to assess the implementation of essential legally and politically regulated policy areas aimed at reducing smoking and exposure to second-hand smoke, as set out in the WHO Framework Convention on Tobacco Control (FCTC). However, the scale does not cover the full range of framework conditions that influence consumers’ decision-making context, for example the agenda of tobacco companies, including their product design as well as pricing and distribution strategies [61]. With regard to the instrument, it should be noted that the current scale are from 2021 and no new ranking has been published since then [27]. Furthermore, the assessment of individual policy areas on the tobacco control scale is not based on a standardised evaluation process, but on the judgements of national experts. It is desirable to further develop the scale in a transparent and evidence-based manner [62].

The consumer price index can be used to compare price trends for individual food groups over recent years. Among these food groups, a distinction can be made between those identified as health-promoting and those whose increased consumption is associated with a higher risk of type 2 diabetes and other NCD [31, 32]. The results regarding the consumer price index show that prices for food and drinks have risen significantly overall over time, particularly for fruit and vegetables, which have a protective association with NCD [33, 63]. There is evidence that price differences and trends in selected food groups influence consumption decisions [23]. As higher food prices can act as a barrier to a healthy diet, particularly for those on lower incomes [30], continuous monitoring of food price trends appears to be advisable.

The health benefits of food are not taken into account in the current tax system [64]. This can make it particularly difficult for low-income groups to maintain a healthy diet [40]. Various professional associations are therefore calling for a tax reduction on health-promoting foods and a tax increase on processed foods with high levels of fat, sugar or salt [58, 65]. Through food taxation, the legislator has the opportunity to specifically influence the prices of health-related goods. This indicator shows the extent to which the health relevance of food is considered in taxation.

The share of prevention expenditure in total health expenditure is relatively low in Germany [66]. Under the heading ‘prevention and health protection’ in the health expenditure billing, in addition to prevention expenditure for NCD, services aimed at infection control are also recorded. Thus, the sharp rise in prevention expenditure relative to total health expenditure in 2021 and 2022 is attributable to additional expenditure on vaccinations and tests during the COVID-19 pandemic. This circumstance limits the comparison over time and must be considered when interpreting the results. Furthermore, many prevention and health promotion measures are delivered outside the health system and are not included in the health expenditure billing. Similarly, no costs are recorded for environmental prevention measures in the health sector and other sectors that go beyond setting-based interventions and are considered promising for reducing risk factors for NCD [58]. Prevention programmes and health promotion measures for NCD cannot currently be comprehensively captured by the health expenditure billing.

The at-risk-of-poverty rate rose between 2005 and 2019; the data shown here, as well as data from the Survey on Income and Living Conditions (EU-SILC) and the Socio-Economic Panel (SOEP), do not initially indicate a further increase for the period from 2020 onwards [44]. Overall, an increase can be observed across the entire period up to 2024 [44]. There are various reasons for the higher and slightly steeper rise in the at-risk-of-poverty rate for women compared to men over time, e.g. a higher rate of part-time work, lower wages (‘gender pay gap’) or gaps in employment history due to the greater burden of unpaid care work, each of which also has corresponding effects on lower pensions. Policy measures to reduce the risk of poverty in the population can have a positive impact on the health of socially disadvantaged groups and help to reduce health inequalities within the population. The indicator ‘at-risk-of-poverty rate’ reflects a subset of contextual factors relating to material or social circumstances. Further indicators for this area should be added to the NCD Surveillance.

A relatively small proportion of distances in Germany are covered actively, i.e. by bicycle or by foot. The expansion of cycle paths and footpaths, particularly by improving their connections to residential and work locations [67], their density and continuity [68], and their safety [69], promotes active mobility among the population. Conclusions regarding environmental factors can only be drawn indirectly from the use of means of transport. It should be investigated which indicators with a more direct link to structures and environmental factors that promote physical activity (e.g. number of green spaces, sports facilities, cycle paths) could be mapped using population-wide data and incorporated into NCD Surveillance in the future.

### 4.3 Conclusion and outlook

The NCD Surveillance at the RKI can help to provide a detailed and comparable picture of indicator-based data on disease prevalence and health outcomes and healthcare provision as well as the largely modifiable risk factors for NCD across Germany over time. These informations can be used by stakeholders in politics and the healthcare sector [6]. Indicators relating to modifiable environmental risk factors are an important component of such surveillance [2].

Currently, so-called ‘commercial determinants of health’ (CDOH) are also gaining importance in public health research; these serve to analyse the effects of political influence as well as the marketing strategies of global economic systems and commercial actors on the health of the population. In particular, there is discussion about how the commercial strategies of companies that produce tobacco, alcohol, highly processed foods or fossil fuels influence key risk factors for NCD [61]. With regard to some of these products, mandatory product labelling and restrictions on advertising and marketing are already being considered as economic contextual factors for health in current projects, such as the Joint Action Prevent NCD [70–74].

The core indicators described in this paper are comparable to indicators relating to contextual factors in other international indicator systems. For example, the Swiss addiction and non-communicable disease monitoring system (MonAM) uses the indicators ‘Active mobility: walking/cycling’ (similar to the proxy indicator ‘use of means transport’) and ‘Regulation of tobacco products’ (similar to the tobacco control scale) [14]. Furthermore, the Public Health Index published by the AOK, a provider of statutory health insurance, in November 2025 highlights the potential for improvement in environmental prevention measures for Germany in a European comparison. When considering measures in the areas of tobacco, alcohol, nutrition and physical activity, Germany ranks 17th out of 18 countries compared to states in Northern and Central Europe [75].

These contextual factor indicators – agreed upon for the first time for the surveillance of diabetes and other NCD in Germany – address some of these aspects and serve as an initial foundation. Going forward, they will be progressively expanded within NCD Surveillance to provide a more comprehensive picture of the conditions in which people live and work.

## Supporting information
