# Supplementary material for: Contextual factors in the surveillance of non-communicable diseases (NCD) in Germany: political, social and environmental indicators
Source: J Health Monit. 2026 Apr 29;11:05. doi: 10.25646/14104 (PMC13137379; doi:10.25646/14104)

Public Health Reporting Germany
Journal of Health Monitoring | **SUPPLEMENTARY MATERIAL**

ROBERT KOCH INSTITUT

Supplement to the publication: Contextual factors in the surveillance of non-communicable diseases (NCD) in Germany: political, social and environmental indicators

Authors:

Laura Neuperdt[1] *
Oktay Tuncer[1] *
Rebekka Mumm[2]
Susanne Jordan[1]
Anne Starker[1]
Kristin Manz[1]
Stephan Müters[1]
Christin Heidemann[1]

Institutions:

[1] Robert Koch-Institute, Department for Epidemiology and Health Monitoring, Berlin, Germany
[2] WIG2 GmbH – Scientific Institute for Health Economics and Health System Research in Germany, Leipzig, Germany

*shared first authorship

Corresponding author:

Laura Neuperdt
E-Mail: NeuperdtL@rki.de

Table 1: Search terms for the bibliographic search

Search block 1: Health Policy Measures

- Maßnahmen + Diabetes + Indikator
- Programme + Diabetes + Indikator
- Prävention + Diabetes + Indikator
- Politik + Diabetes + Indikator
- Gesundheitspolitische Maßnahmen + Diabetes + Indikator
- Präventionsprogramme + Diabetes + Indikator
- Prävention + Diabetes + Evaluation
- Präventionsmaßnahmen + Diabetes
- Primärprävention + Diabetes
- Prevention + diabetes + indicator
- Prevention + programme + diabetes + indicator
- Primary prevention + diabetes

Search block 2: Employment and Social Affairs

- Betriebliche Gesundheitsförderung + Diabetes + Indikator
- Arbeit + Diabetes + Indikator
- Beruf + Diabetes + Indikator
- Sozial + Diabetes + Indikator
- Arbeit + Diabetes + Prävention + Indikator
- Beruf + Diabetes + Prävention + Indikator
- Sozial + Diabetes + Prävention + Indikator
- Health promotion + diabetes + work + indicator
- Diabetes + job + indicator
- Diabetes + work + indicator
- Diabetes + society + indicator
- Diabetes + social + determinants of health + indicator
- Diabetes + job + prevention + indicator
- Diabetes + work + prevention + indicator
- Diabetes + society + prevention + indicator

Search block 3: Built and Physical Environment

- Bebaute Umwelt + Gesundheit + Indikator
- Bebaute Umwelt + Diabetes + Indikator
- Adipogene Umwelt + Indikator
- Built environment + health + indicator
- Built environment + diabetes + indicator
- Obesogenic environment + indicator

Search block 4: Cross-thematic indicators

- Kontextfaktoren + Gesundheit + Indikator
- Kontextfaktoren + Diabetes + Indikator
- Contextual factors + health + indicator
- Context indicators + health + indicator

Table 2: List of the 19 indicators for the two-stage Delphi procedure with the Scientific Advisory Board of the Diabetes Surveillance

Indicator	Description
At-risk-of-poverty rate	Proportion of people whose equivalised disposable income is less than 60% of the median equivalised disposable income of the population (in private households) (=at-risk-of-poverty threshold)
Unemployment rate	Proportion of unemployed people as a percentage of all civilian workers (employed + unemployed) in %
Professional activity	Proportion of people currently engaged in a specific professional activity as their main occupation (main occupational groups (2-digit codes according to KldB2010) broken down by occupational segments)
Net household income	Average net equivalent income in € per inhabitant
Workload	Proportion of the working population who frequently push themselves to the limits of their capacity at work or work under intense time and performance pressure
Shift work	Proportion of people working shifts (broken down by shift type (early shift, late shift, night shift, rotating shift))
Availability of workplace health promotion	Number of locations/workplaces offering workplace health promotion
Utilisation of workplace health promotion	Number of people directly reached by workplace health promotion

Air quality – particulate matter	Annual average (in µg/m³) of particulate matter concentrations (PM10 or PM2.5)
Number of days on which PM10 limits were exceeded	Number of days on which the PM10 limit value was exceeded (= 50 µg/m³)
Noise pollution	Proportion of the total population affected where the 24-hour day-evening-night noise index (Lden) exceeds 65 dB
Availability of sports, leisure and recreational areas	Proportion of sports, leisure and recreational areas as a percentage of the settlement area (in km²)
Use of means of transport	Modal split of use of means of transport (in %)
Tobacco control	Scale for assessing a country's tobacco control policy, based on the Tobacco Control Scale
Prevention expenditure	Proportion of prevention expenditure in total health expenditure per capita
Advertising expenditure	Level of commitment to generally applicable restrictions on the marketing of food to children, young people and adults a) on the web/social media, b) on television, c) in print media and d) on packaging<br>or: Advertising expenditure in € for selected food products
Consumer price index	Consumer price indices (CPI) for a) fruit, b) vegetables, c) sweets, d) ready-made meals, e) soft drinks, and f) red meat
Existence of reformulation strategies	Degree of commitment to reformulation targets for a) trans fats, b) saturated fats, c) sugar and d) salt

Food taxation/subsidies	Tax burden in cents per 100 cents of food price for a) fruit and vegetables, b) sugary soft drinks, c) sugar, d) frozen pizza, e) mineral water, and f) red meat

Figure 1: Tax burden in cents per 100 cents between 1990 and 2024. Source: Value Added Tax Act [35]

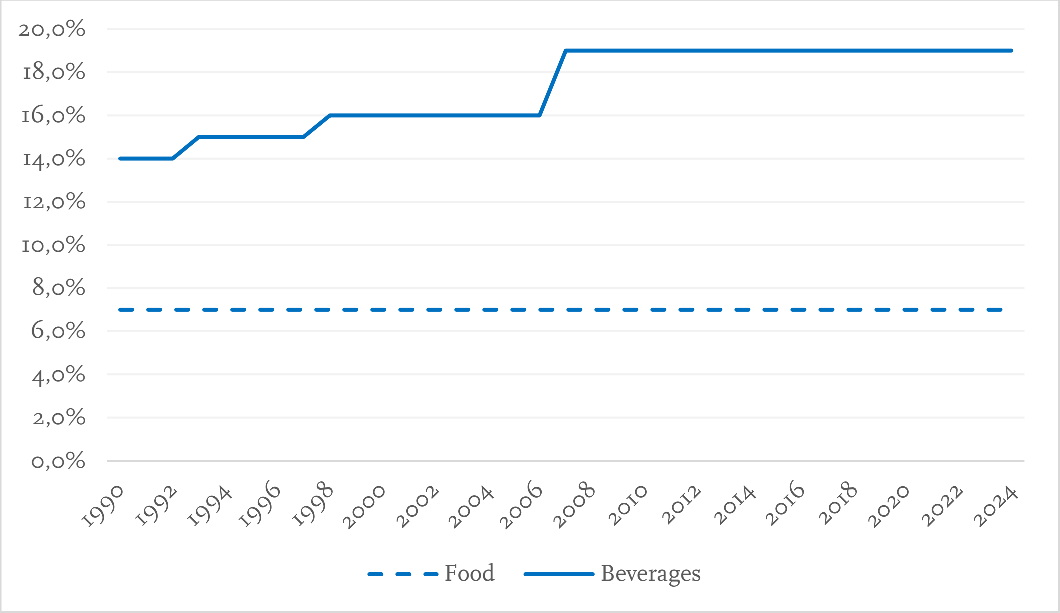

Figure 2: Trend in prevention expenditure between 1992 and 2023. Source: Health Expenditure Accounts [37]

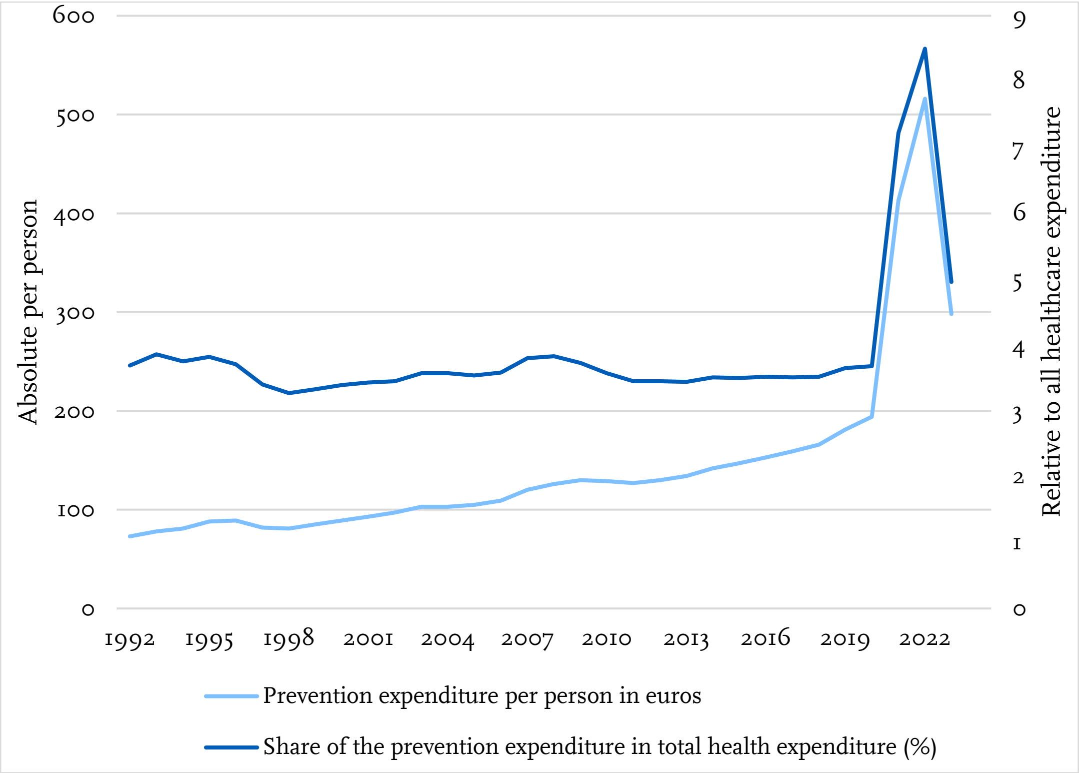

Supplement: Supplementary file 1 [file Supplement-2026-01.zip › Supplement-2026-01.pdf]
